# A revision of *Laeliichthys ancestralis* Santos, 1985 (Teleostei: Osteoglossomorpha) from the Lower Cretaceous of Brazil: Phylogenetic relationships and biogeographical implications

**DOI:** 10.1371/journal.pone.0241009

**Published:** 2020-10-29

**Authors:** Paulo M. Brito, Francisco J. Figueiredo, Maria Eduarda C. Leal

**Affiliations:** 1 Departamento de Zoologia, Instituto de Biologia (IBRAG), Universidade do Estado do Rio de Janeiro, Rio de Janeiro, RJ, Brazil; 2 Faculty of Health, Aarhus University, Aarhus, Denmark; Università degli Studi di Torino, ITALY

## Abstract

A re-description of the freshwater, Early Cretaceous osteoglossomorph *Laeliichthys ancestralis* Santos, 1985, from the Sanfranciscana Basin of Brazil, is provided. New anatomical details and a revised diagnosis, as well as a new phylogeny are presented. A phylogenetic analysis places this taxon within the Osteoglossomorpha most likely as a member of the Notopteroidei. Within this clade *Laeliichthys* is the sister-taxon of the Notopteridae. The subfamily Laeliichthyinae is elevated to family rank. The revised phylogenetic position revealed in this study has important consequences on the biogeography of Notopteroidei as it extends their distribution to western Gondwana, prior to the separation of South America and Africa, and extends the evolutionary origins of notopteroid lineages by at least ~27 Myr before their first appearance in the fossil record.

## Introduction

The species *Laeliichthys ancestralis* Santos, 1985 [1] derives from 125 million years old deposits of the Quiricó Formation, Sanfranciscana Basin, Brazil (Lower Cretaceous- Barremian) and is one of the oldest known members of the Osteoglossomorpha, the so-called bony-tongue fishes. Osteoglossomorphs are primarily freshwater fishes [2–4], with a long and diverse fossil record, including only a few species from marine deposits [5–10].

Presently osteoglossomorphs comprise six extant families [3] and have phylogenetic importance due to their basal position among the Teleostei. They have been considered as the most basal lineage among living teleosts [11–13], although more recent studies have questioned this position, hypothesizing that elopomorphs are the most basal extant teleosts [14–16].

*Laeliichthys ancestralis* was first illustrated and briefly discussed by Taverne [17], who considered it to be closely related to the extant genus *Heterotis*. A few years later, Santos [1] named this species and provided a formal description, assigning it to the family Arapaimidae, within its own subfamily Laeliichthyinae. Subsequently, *Laeliichthys* has been cited or incorporated within the phylogenetic analyses of diverse authors who considered it as a stem osteoglossid [18] or as a stem Arapaiminae (= Heterotidinae) [6,19–25]. These analyses were all based on Santos’ original description [2]. The first concerns regarding the position of *Laeliichthys* among the Osteoglossoidei were raised by Meunier & Brito [26] who, studying the morphology and the histology of the scales in basal teleosts, showed that the scales of *Laeliichthys* were highly distinct from those of both arapaimids and osteoglossids.

Therefore, *Laeliichthys* is of particular interest not only because of its age but also because understanding its phylogenetic position may have important implications for understanding the evolution and biogeography of osteoglossomorphs in general and in the western part of Gondwana in particular. Further preparation of the original material of *Laeliichthys ancestralis* as well as the use of new imaging techniques led us to reveal new aspects of the morphology of this taxon. This study provides a re-description of this exquisitely preserved taxon, and re-evaluates its phylogenetic position.

### Type locality and geological age

**Fig 1:**
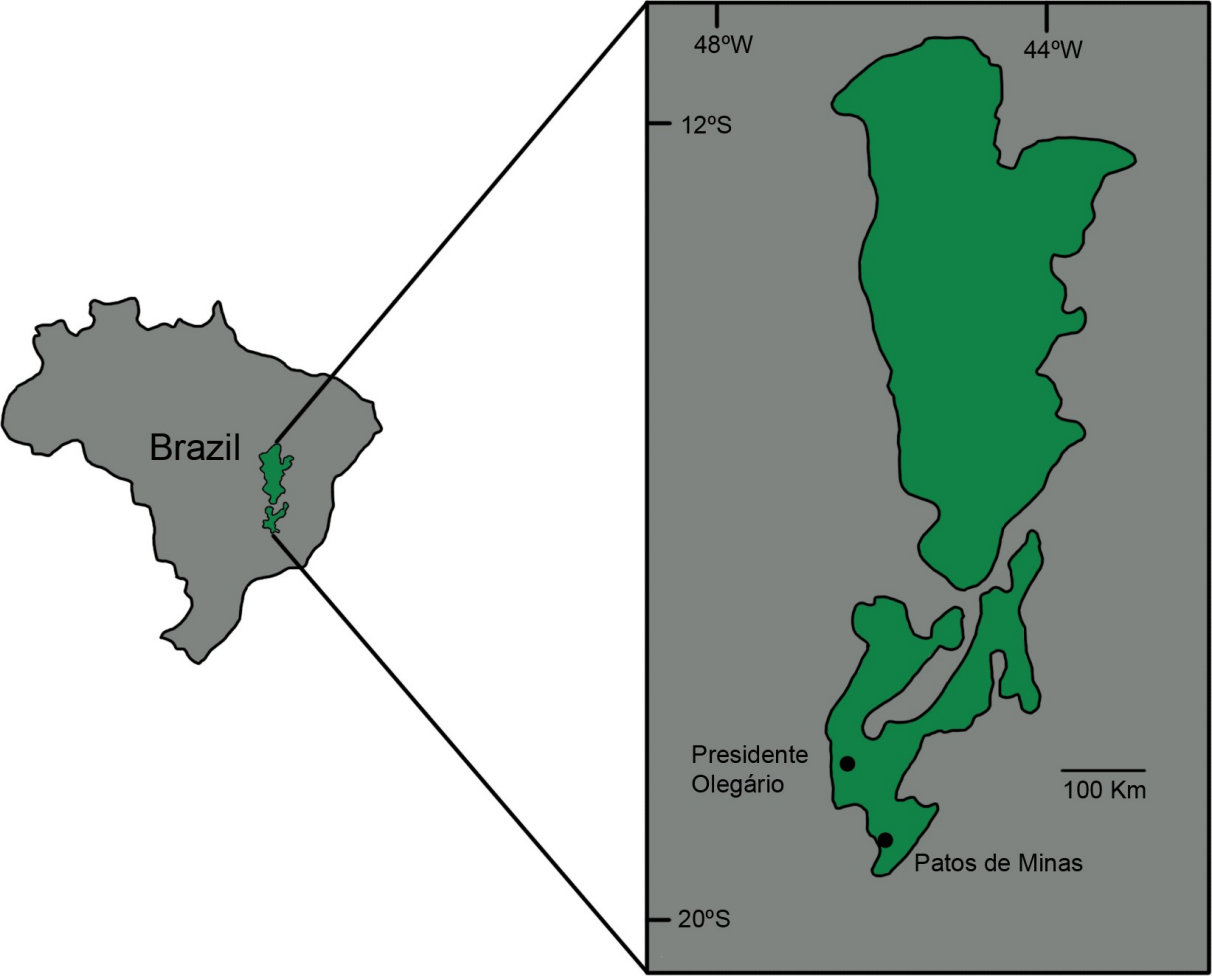
Map of the Sanfranciscana Basin.

The type locality exposes Early Cretaceous, bituminous shales and laminated carbonates (= fish-levels) of the upper part of the Quiricó Formation, in the Sanfranciscana Basin, State of Minas Gerais, in the quarries of the São José do Geribá farmstead, near the town of Patos de Minas, southeastern Brazil (Fig 1). *Laeliichthys* is always found associated with the gonorynchiform *Dastilbe crandalli* in these levels [27,28].

The minimal age of the fish-levels of the Quiricó Formation is generally regarded as late Barremian based mainly on palynological dates, and lies within the *Transitoripollis (Tucanopollis) crisopolensis* palinozone [28–31]. A revision of this horizon [32] revealed the presence of the palinomorph *Tucanopollis annulatus*, a taxon described from the upper Six Hills Formation of the Dakhla oasis in Egypt [33]. Therefore, the age of the fish-level from the Quiricó Formation is most likely late Barremian (~121 Mya).

## Material and methods

### Material

All the specimens described here (specimen numbers: UERJ-DZ01, UERJ-DZ02, UERJ-DZ03, UERJ-DZ04, UERJ-DZ05, UERJ-DZ06, UERJ-DZ17, UERJ-DZ18, UERJ-DZ19, DGM-1158.P, and DGM-1159.P.) were collected in the field by researchers of the Universidade do Estado do Rio de Janeiro (UERJ) and the Departamento Nacional de Produção Mineral (DNPM) and come from the same general locality near the town of Patos de Minas, Minas Gerais State, southeastern Brazil. All the specimens used in this study are permanently housed in public collections in Brazil (Universidade do Estado do Rio de Janeiro and Departamento Nacional de Produção Mineral).

No specific permits were required for the described field work. In Brazil, the unique obligation we have is to contact the Departamento Nacional de Produção Mineral (DNPM–The Brazilian Geological Survey) explaining that we are doing fieldwork and after that to curate fossils in an official public collection, what is the case of the studied specimens deposited in the collection of the Universidade do Estado do Rio de Janeiro and the Departamento Nacional de ProduçãoMineral.

### Terminology

We employed the traditional anatomical and directional terms used for describing actinopterygian cranial osteology [34,35]. For the postcranial anatomy we used the terminology proposed by [36].

### Institutional abbreviations

DGM Divisão de Geologia e Mineralogia, Departamento Nacional de Produção Mineral, Rio de Janeiro, Brazil; UERJ Universidade do Estado do Rio de Janeiro, Rio de Janeiro, Brazil.

### Phylogenetic analysis

The phylogenetic analysis was performed using a modified data matrix from Wilson & Murray [37], which constitutes the most comprehensive matrix for Osteoglossomorpha; this data matrix was originally built using characters proposed by Hilton [38] and Li et al. [39].

The matrix was only modified by including *Laeliichthys ancestralis*. The character states for *Laeliichthys* are presented in the S1 Fig. The phylogenetic analysis was run using the parsimony method in PAUP 4b10 [40] under ACCTRAN optimization. All characters were equally weighted and treated as unordered.

### Systematic palaeontology

Superorder Osteoglossomorpha Greenwood *et al*., 1966 [41].
Order Osteoglossiformes Regan 1909 [42].
Suborder Notopteroidei Jordan, 1923 [43].
Family Laeliichthyidae New.
Type genus—*Laeliichthys* Santos, 1985 [1] (family monotypic).

#### Family diagnosis

Laeliichthyidae differs from all other families within Notopteroidei (here including Notopteridae, Mormyridae, Gymnarchidae and the genus *Palaeonotopterus*) by the following characters: nasal with semi-triangular outline, broad posteriorly with sensory canal opening in a groove and; infraorbitals completely cover the palatoquadrate behind and below the orbit; first pectoral fin ray enlarged and extremely long.

Genus *Laeliichthys* Santos, 1985 [1]

#### Generic diagnosis

As for the only known species below.

*Laeliichthys ancestralis* Santos, 1985 [1]

**Fig 2:**
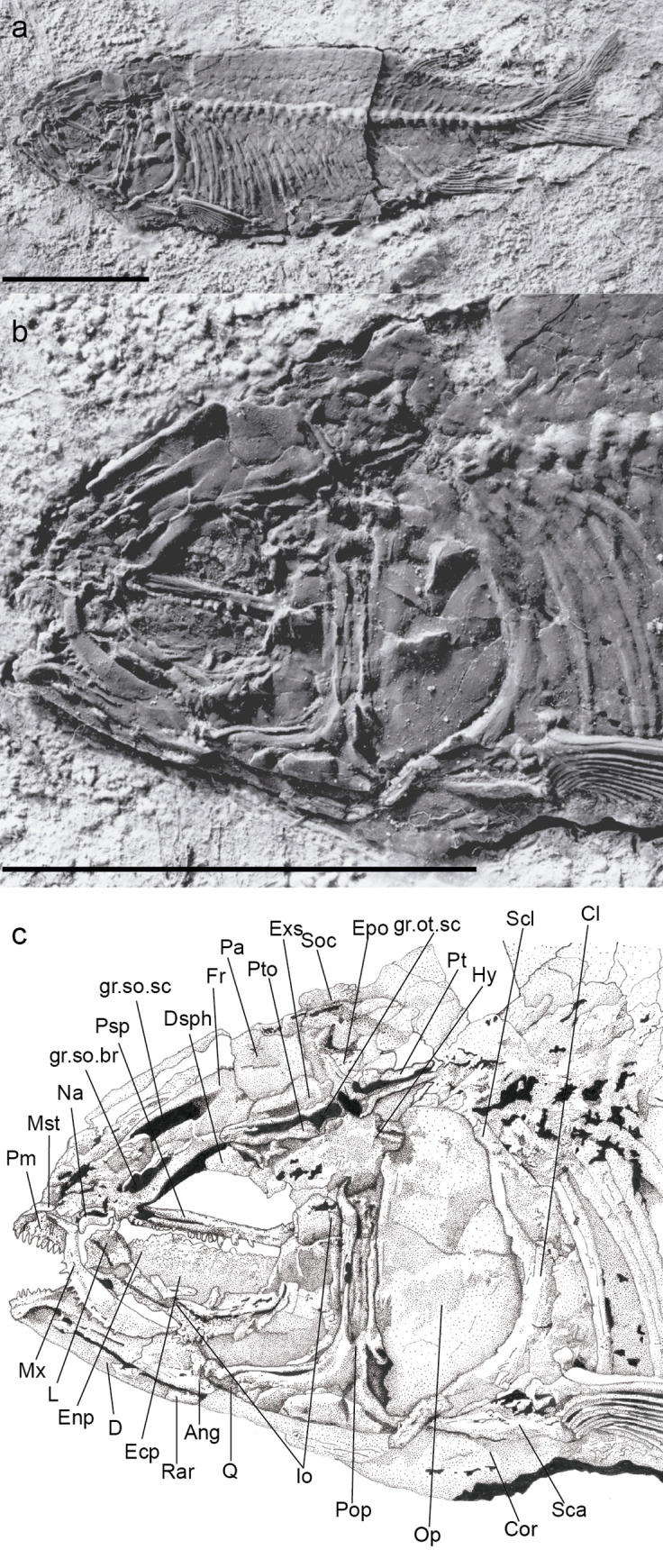
*Laeliichthys ancestralis* Santos, 1985 [1]. Holotype UERJ-DZ03. **A,** Complete specimen; **B,** photograph of the head region; **C,** anatomical interpretations. Abbreviations: Ang, angular; Cl, cleithrum; Cor, coracoid; D, dentary; Dsph, dermosphenotic; Ecp, ectopterygoid; Epo, epiotic; Enp, endopterygoid; Exs, extrascapular; Fr, frontal; gr.ot.sc, groove for otic sensory canal; gr.so.br, groove for supraorbital branch of otic sensory canal; gr.so.sc, groove for supraorbital sensory canal; Hy, hyomandibula; Io, infraorbital; L, lacrimal; Mst, mesethmoid; Mx, maxilla; Na, nasal; Op, opercle; Pa, parietal; Pm, premaxilla; Pop, preopercle; Psp, parasphenoid; Pto, pterotic; Pt, posttemporal; Q, quadrate; Rar, retroarticular; Sca, scapula; Scl, supracleithrum. Scale bar equals 15 mm.

Holotype. UERJ-DZ03. An almost complete specimen, displaying its left side (Fig 2).

Additional Material. UERJ-DZ01, UERJ-DZ02, UERJ-DZ04, UERJ-DZ05, UERJ-DZ06, UERJ-DZ17, UERJ-DZ18, UERJ-DZ19, DGM-1158.P, DGM-1159.P.

#### Emended diagnosis

Small sized, elongate bodied osteoglossomorph with semi-triangular nasal meeting its antimere in the midline of the skull; lateral line sensory canal partially open in a groove; frontal with constriction over the orbit; supraorbital sensory canal running longitudinally in a gutter-like groove; smaller groove for the supraorbital branch of the otic canal on the margin of the frontal continuing through the pterotic; Lateral line not piercing the supracleithrum; parietal subrectangular with supratemporal comissure; extrascapular reduced and irregularly shaped, overlying the lateral surface of the parietal and pterotic; preopercle sensory canal opening by a groove on the dorsal limb and large pores ventrally; opercle hypertrophied with a flattened dorsal surface; subopercle and interopercle absents; palatoquadrate area, behind and below orbit, covered by infraorbitals; infraorbital canal running in a narrow gutter in the antorbital and first three infraorbitals but closed in the fourth infraorbital and dermosphenotic; the mandibular canal open in a groove; basihyal toothplate with ventrally directed processes; leading pectoral ray strong and long, extending beyond the origin of the pelvic fin; dorsal and anal fins triangular and located posteriorly in the body; caudal fin with forked outline and lobes of equal size; caudal endoskeleton comprising two preural and two ural centra; preural centra 2 and 1 carry, each one, a full length neural spines; first ural centrum with complete neural spine; scales with no reticulate furrows.

### Anatomical description

This is a small sized osteoglossiform; the holotype is 85 mm total length (TL) and about 76 mm standard length (SL). Considering the good condition of several specimens, the cranial measurements taken on the largest individual (UERJ-DZ02) provide a relatively accurate estimate of the total length of this specimen of about 113 mm. The deepest part of the body is constant between the pectoral and the posterior part of the pelvic fin. The head, including the opercular series, measures approximately 25% of the total length. The dorsal fin is located along the posterior third of the body, and its origin is slightly anterior of the anal fin. The pelvic fin is located halfway between the pectoral and the anal fin. The caudal fin is deeply forked.

#### Skull roof

**Fig 3:**
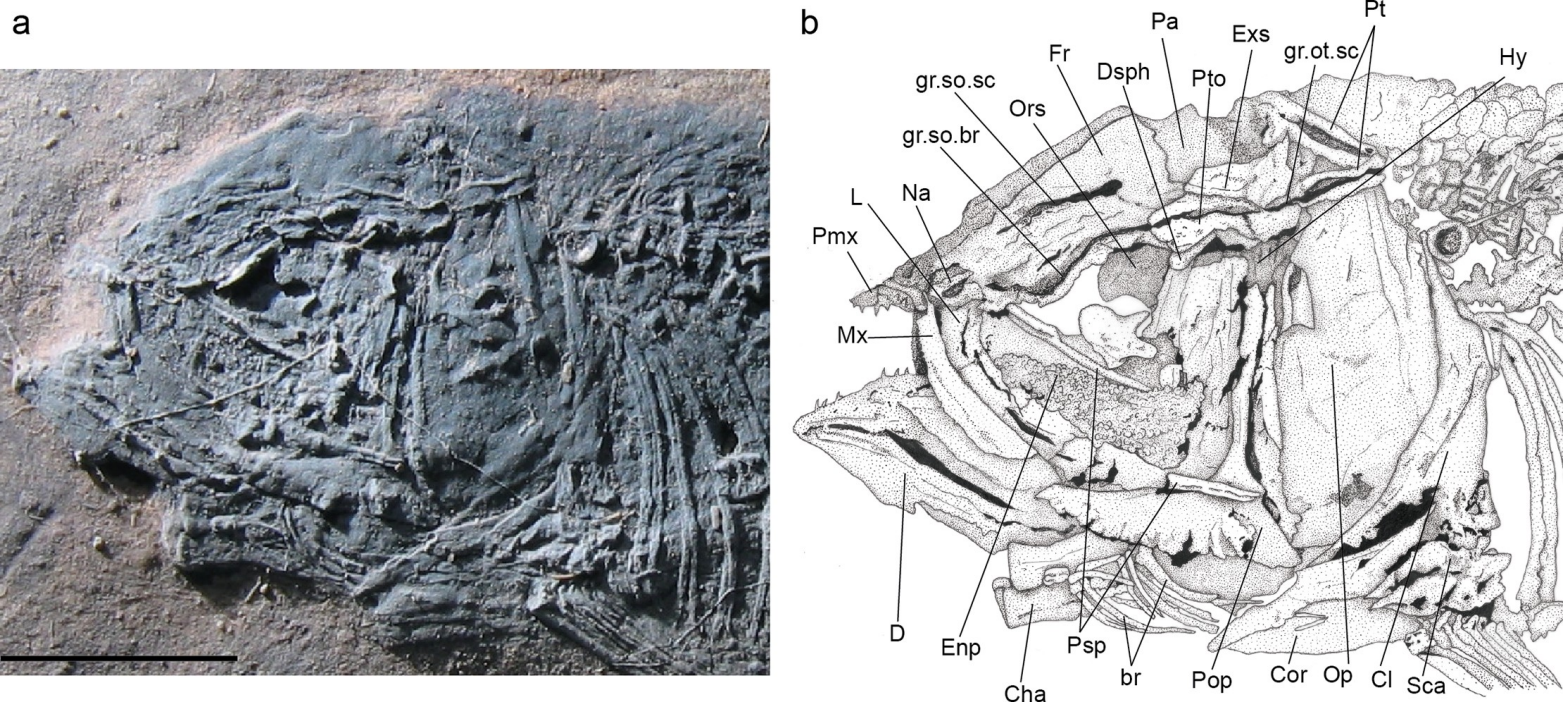
*Laeliichthys ancestralis* Santos, 1985 [1]. Specimen DGM-1158.P. **A,** photograph of the head region; **B,** anatomical interpretations. Abbreviations: br, branchiostegals; Cha, anterior ceratohyal; Cl, cleithrum; Cor, coracoid; D, dentary; Dsph, dermosphenotic; Enp, endopterygoid; Fr, frontal; gr.ot.sc, groove for otic sensory canal; gr.so.br, groove for supraorbital branch of otic sensory canal; gr.so.sc, groove for supraorbital sensory canal; Hy, hyomandibula; L, lacrimal; Mx, maxilla; Na, nasal; Op, opercle; Ors, orbitosphenoid; Pa, parietal; Pmx, premaxilla; Pop, preopercle; Psp, parasphenoid; Pt, posttemporal; Pto, pterotic; Sca, scapula. Scale bar equals 5 mm.

**Fig 4:**
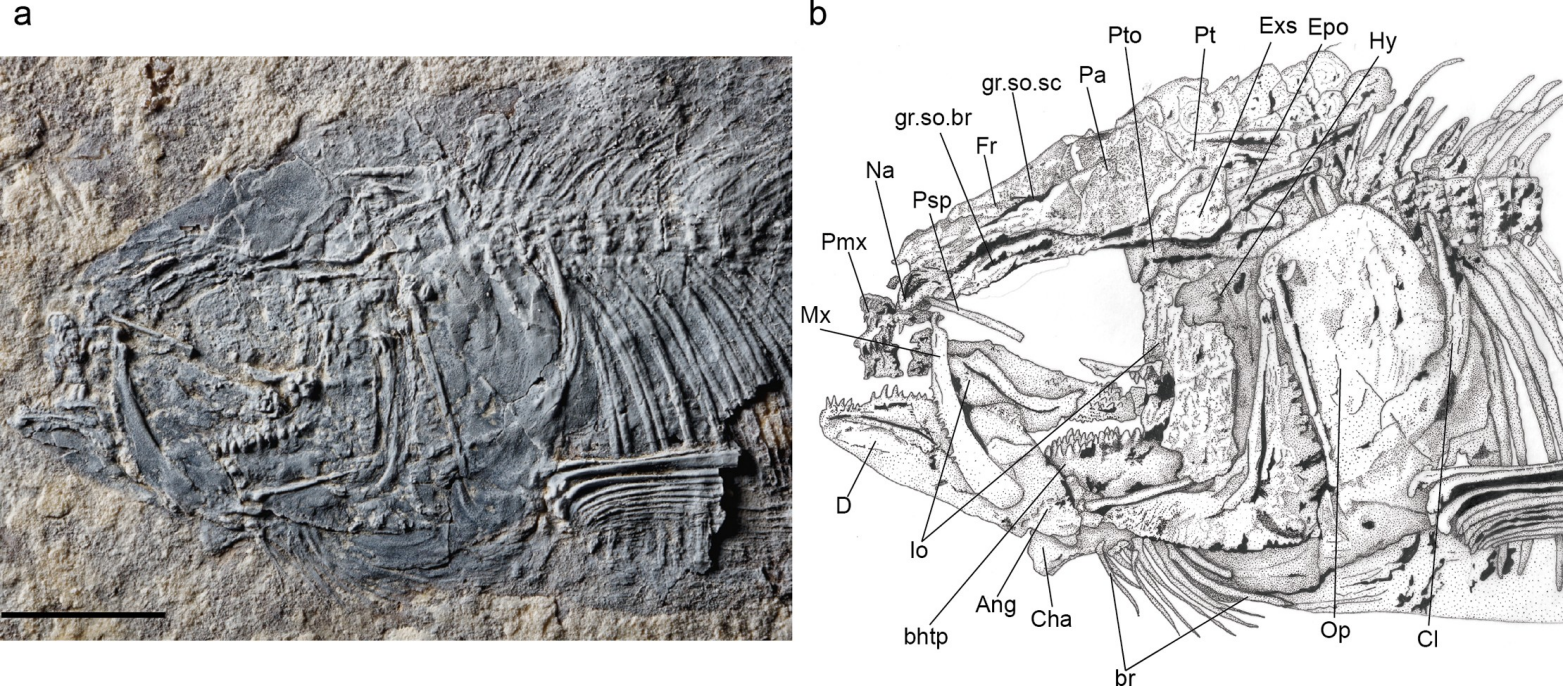
*Laeliichthys ancestralis* Santos, 1985 [1]. Specimen DGM-1159.P. A, photograph of the head region; **B,** anatomical interpretations. Abbreviations: Ang, angular; br, branchiostegals; bhtp, basihyal toothplate; Cha, anterior ceratohyal; Cl, cleithrum; D, dentary; Epo, epiotic; Exs, extrascapular; Fr, frontal; gr.so.br, groove for supraorbital branch of otic sensory canal; gr.so.sc, groove for supraorbital sensory canal; Hy, hyomandibula; Io, infraorbital; Mx, maxilla; Na, nasal; Op, opercle; Pa, parietal; Pmx, premaxilla; Psp, parasphenoid; Pt, posttemporal; Pto, pterotic. Scale bar equals 5 mm.

**Fig 5:**
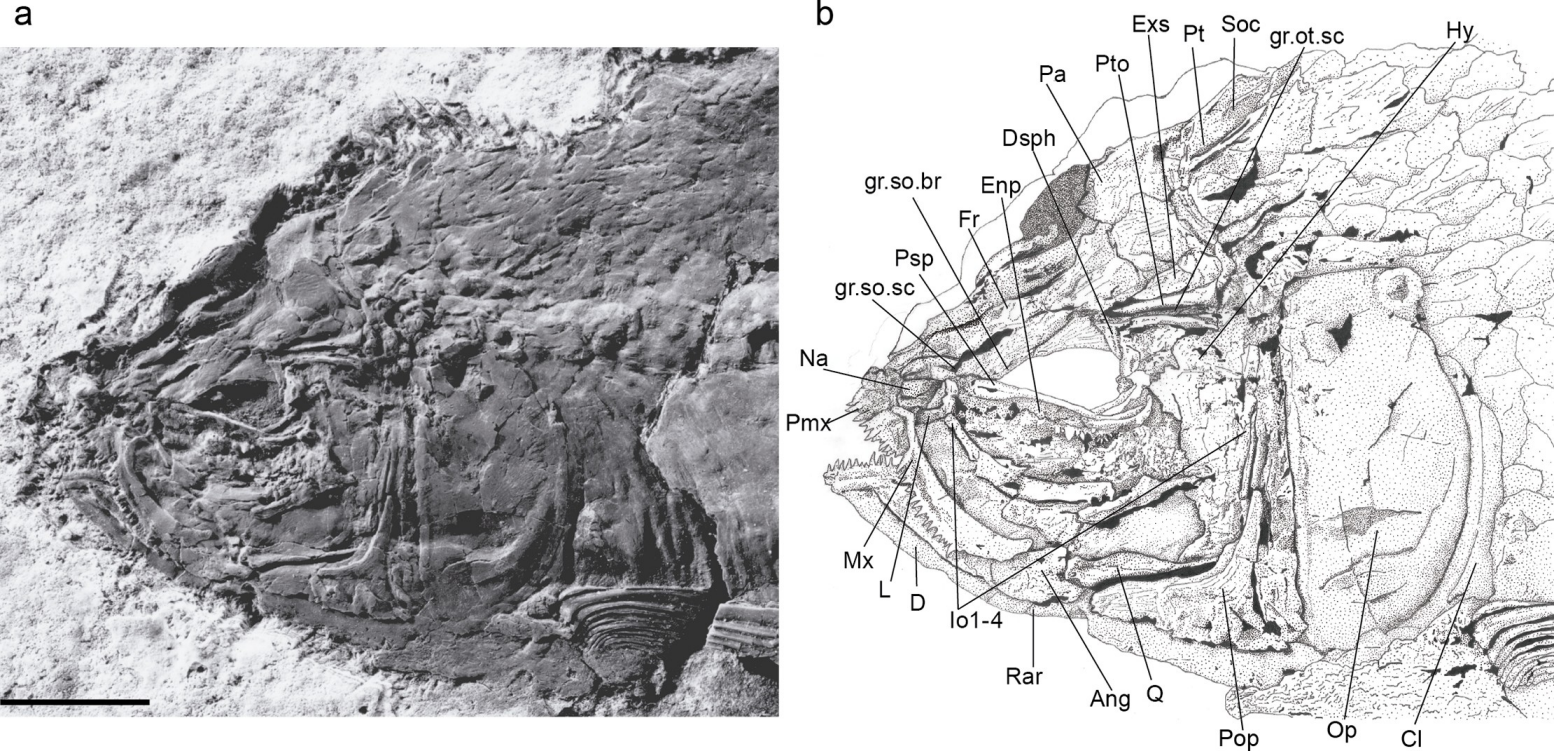
*Laeliichthys ancestralis* Santos, 1985 [1]. Specimen UERJ-DZ 002. **A,** photograph of the head region; **B,** anatomical interpretations. Abbreviations: Ang, angular; Cl, cleithrum; D, dentary; Dsph, dermosphenotic; Enp, endopterygoid; Exs, extrascapular; Fr, frontal; gr.ot.sc, groove for otic sensory canal; gr.so.br, groove for supraorbital branch of otic sensory canal; gr.so.sc, groove for supraorbital sensory canal; Hy, hyomandibula; Io 1–4, infraorbitals 1 to 4; L, lacrimal; Mx, maxilla; Na, nasal; Op, opercle; Pa, parietal; Pmx, premaxilla; Pop, preopercle; Psp, parasphenoid; Pt, posttemporal; Pto, pterotic; Q, quadrate; Rar, retroarticular Soc, supraoccipital. Scale bar equals 5 mm.

The skull roof is partially preserved, although somewhat distorted, on specimens UERJ-DZ02, UERJ-DZ03, DGM-1158.P, and DGM-1159.P (Figs 2–5).

The nasal has a sub-triangular outline, slightly curved ventrally anteriorly and is broad posteriorly. It meets its antimere in the midline of the skull and carries the anterior part of the lateral line sensory canal that extends through its posterior portion as an open groove. The anterior tip of the nasals may be separated by the mesethmoid. The mesethmoid is preserved only on specimen UERJ-DZ03 (Fig 2), where its anterior part is slightly narrower than its posterior part.

**Fig 6:**
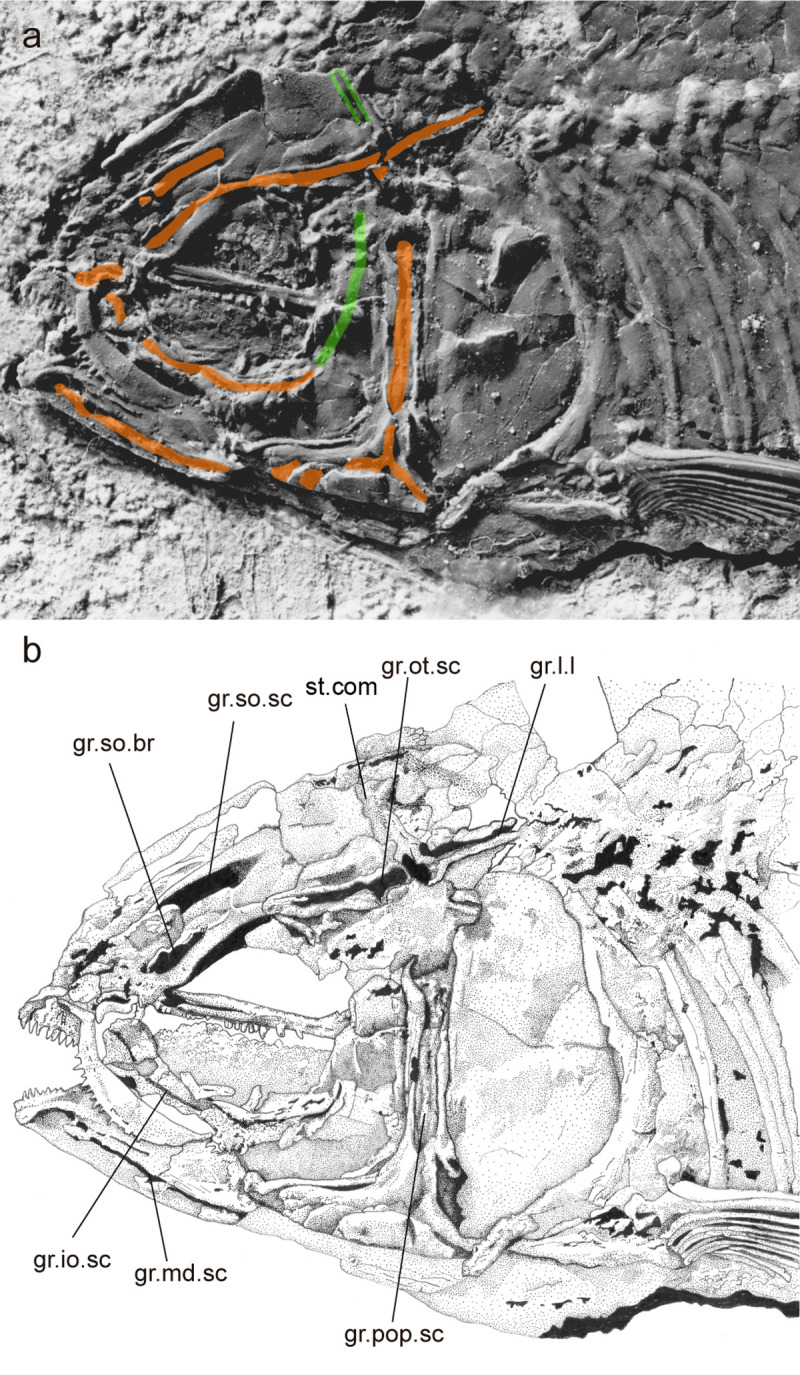
*Laeliichthys ancestralis* Santos, 1985 [1]. Holotype UERJ-DZ03, sensory canals. Abbreviations: gr.io.sc, groove for infraorbital sensory canal; gr.md.sc, groove for mandibular sensory canal; gr.pop.sc, groove for preopercular sensory canal; gr.ot.sc, groove for otic sensory canal; gr.so.br, groove for supraorbital branch of otic sensory canal; gr.so.sc, groove for supraorbital sensory canal; st.com, supratemporal commissure. Traces in orange represent the sensory canals running in grooves; in green sensory canal, or segments of canal, that lies entirely within a bony tube.

The frontal is the longest element of the dermal skull, having a length approximately 1,5 times that of the parietal, and extending from the nasal posteriorly to the parietal and pterotic (Figs 2–5). The bone is somewhat constricted over the orbit. The suture between the frontals is almost straight. The partially roofed supraorbital sensory canal extends longitudinally on a prominent gutter-like groove that ends in the posterior part of the bone. Lateral to this canal, close to the lateral margin of the bone, there is a smaller groove for the supraorbital branch of the otic canal, that continues through the pterotic (Figs 2–6). This canal and the supraorbital canal lie side by side in parallel grooves separated by a bony ridge.

The parietal is subrectangular and well-developed (Figs 2–5). Its lateral border is almost linear and contacts the extrascapular. The supratemporal commissure passes through the parietals (Fig 6) and lies entirely within a bony tube in the posterior part of the bone. Posteriorly both parietals partially cover the front of the supraoccipital. The extrascapular is a nearly triangular bone and overlies the lateral surface of the parietals and the pterotics (Figs 2–5), as in *Xenomystus* and *Papyrocranus* [38,44,45]. This bone extends forward to the frontals.

Lateral to the parietal and partially covered by the extrascapular is the long and somewhat rectangular pterotic. The otic sensory canal lies in a groove that extends posteriorly until the rear of the bone, where it continues in the post-temporal. In its posterior third, the otic sensory canal groove bifurcates joining laterally the groove for the preopercle sensory canals (Fig 6). Ventrally, the pterotic roofs the majority of the hyomandibular facet.

#### Braincase

Some neurocranial elements could be observed, it being possible to identify a few bones, visible in the orbital region in specimens UERJ-DZ02 and DGM-1158.P (in addition to the pterotic and the epiotic cited above). The orbitosphenoid is a large, unpaired bone, displaying the usual "V" shape. The basisphenoid is partially preserved and only its median basal peduncle, resting on the dorsal surface of the parasphenoid can be seen.

The supraoccipital is a somewhat triangular, bearing a small supraoccipital crest (Figs 2 and 5). Lateral to the supraoccipital the epiotic is a small triangular bone located above the exoccipital.

The parasphenoid is a long bone that crosses the middle of the orbit (Fig 2), bearing robust conical teeth on its ventral margin. These teeth lie in a single row within the orbital region and in two rows below the basipterygoid process. The basipterygoid process is reduced, similar to the process found in mormyroids.

#### Circumorbital bones

The orbit is large and its length is approximately one-third of the head length. There are six circumorbital bones: the antorbital, four infraorbitals and the dermosphenotic. The antorbital is a small, rectangular bone, lying on the antero-dorsal edge of the maxilla. It contacts the nasal dorsally and the first infraorbital posteriorly.

The first infraorbital (= lacrimal) is longer than deep, and has a rectangular-shape in lateral view (Figs 2 and 3). The second infraorbital is larger than the first one and becomes deeper posteriorly; these two bones are placed on the ventral margin of the orbit.

The third infraorbital is positioned on the postero-ventral corner of the orbit. It is an expanded bone and has a size somewhat equivalent to that of the second infraorbital, covering the metapterygoid and part of the quadrate. The fourth infraorbital is the dorsalmost of the series. It covers the hyomandibula and dorsally reaching the dermosphenotic. The dermosphenotic is a subtriangular bone that contacts the anterolateral edge of the dermopterotic.

The infraorbital canal runs in a narrow gutter along the upper third of the first three infraorbital bones and seems to be closed in an elongated tube in the fourth infraorbital and the dermosphenotic (Fig 6).

#### Jaws

The upper jaw consists of paired premaxillae and maxillae (Figs 2–5). The premaxilla consists in an anterior, laterally elongated oral border, bearing nine conical teeth, (Figs 2, 4 and 5).

The maxilla is elongate and increases in depth posteriorly (Figs 2–5). Its anterior portion curves slightly inwards, forming an elongate narrow process that articulates with the premaxilla. As in the premaxilla, the oral border of the maxilla bears a single row of conical teeth. These teeth are of similar size to those on the premaxilla. Contrary to the original description [1] there is no supramaxilla in *L*. *ancestralis*.

The lower jaw consists of the dentary, angular (fused to articular), and retroarticular. The dentary forms the anterior part of the mandible. The symphysis is medially curved and the bone increases in height posteriorly (Figs 2 and 4). The anterior portion of the oral margin of the dentary bears about 10 conical teeth of approximately equal length. Posteriorly, the dentary sutures with the angular, that appears to have fused with the articular, while the angular sutures posteriorly with the small retroarticular (Figs 2 and 4). The mandibular sensory canal is contained within a gutter in the dentary and angular.

#### Palato-pterygo-quadrate arch

The endopterygoid is elongate and forms most of the ventral wall of the orbit. It has a straight upper edge and an arched lower edge, bearing a broad tooth patch with numerous densely set conical teeth on its inner surface (Figs 2, 3 and 5).

The ectopterygoid lies to the external ventral side of the endopterygoid (Fig 2). It is slender and bears teeth. The form of the metapterygoid is still unclear as it is usually concealed by the posterior infraorbitals. However, on specimen UERJ-DZ01 this bone is visible and appears more or less square, perhaps slightly longer than high.

The quadrate is a fan-shaped bone with a large and elongated ventral process (Figs 2 and 5). The anteroventral end of the quadrate bears a process that articulates with the angulo-articular.

#### Hyoid arch, gill arches, and branchiostegals

The symplectic is small and located in a notch between the main body of the quadrate and the ventral process of this bone. The hyomandibula is preserved in its natural position in specimens UERJ-DZ02, UERJ-DZ03, and DGM-1158.P (Figs 2, 3 and 5). It is robust with a large dorsal head, an anteroventral process, and a small ventral process. The hyomandibula articulates with the neurocranium via a facet comprising the dermosphenotic and pterotic.

The anterior ceratohyal is hour-glass-shaped, with the anterior part narrow and small and the posterior part broad and large (Figs 3 and 4). The posterior ceratohyal cannot be observed. At least nine branchiostegals are present in DGM-1558.P and DGM-1559.P. These elements are thin and are of constant width (Figs 3 and 4).

A basihyal tooth plate is well preserved in specimen DGM-1559.P. It is a well-developed plate, with curved caniniform teeth located on the margin (Fig 4).

#### Preopercle and opercular series

The preopercle is well-developed and divided into a vertical dorsal ramus and an expanded ventral part (Figs 2–5). The sensory canal extends down along the anterior margin of the vertical limb, and then turns anteriorly along the dorsal margin of the horizontal limb until it enters the angular. The sensory canal opens into a groove on the dorsal limb with large pores arranged in branches ventrally (Fig 6).

The opercle is almost twice as deep as it is long. Its anterior border is somewhat straight, perpendicular to the axis of the head. Its dorsal margin is flattened. Although Santos [1] described a subopercle and an interopercle, none of these bones could be observed in the examined material.

#### Paired fins and girdles

The dermal elements of the pectoral girdle are preserved in nearly all specimens. The posttemporal is a forked bone (Figs 3 and 4). The dorsal arm is just slightly longer than the ventral arm. The ventral arm carries the lateral sensory canal. Underlying the posterolateral corner of the posttemporal is the anterodorsal tip of the supracleithrum. The supracleithrum is elongated with a tapered dorsal tip. It is not perforated by the sensory canal of the lateral line as this canal extends directly from the posttemporal to the lateral line scales. The cleithrum is somewhat L-shaped with its anteriorly lower arm underlying the opercle; the bone has a curved external surface. A broad medial flange, described by Cavin and Forey [45] for *Palaeonotopterus* and also found in mormyrids, doesn't seem to be present in *Laeliichthys*.

The scapulocoracoid articulates just behind the ventral part of the dorsal arm of the cleithrum. The pectoral fin articulates with the somewhat round propterygial ossification and the distal radials. The pectoral fin contains ten rays. The leading ray is unbranched but segmented distally, and quite strong and long, extending beyond the origin of the pelvic fin, a pattern also found in *Singida*, *Phareodus*, *Paralycoptera*, and extant osteoglossins [46]. The other nine rays are branched and segmented.

The pelvic girdle of *Laeliichthys* is a slender element, the pelvic bone. Articulating with it are six or seven fin rays, all branching distally. Like in the pectoral fin, the anterior pelvic rays are the widest.

#### Dorsal and anal fins

**Fig 7:**
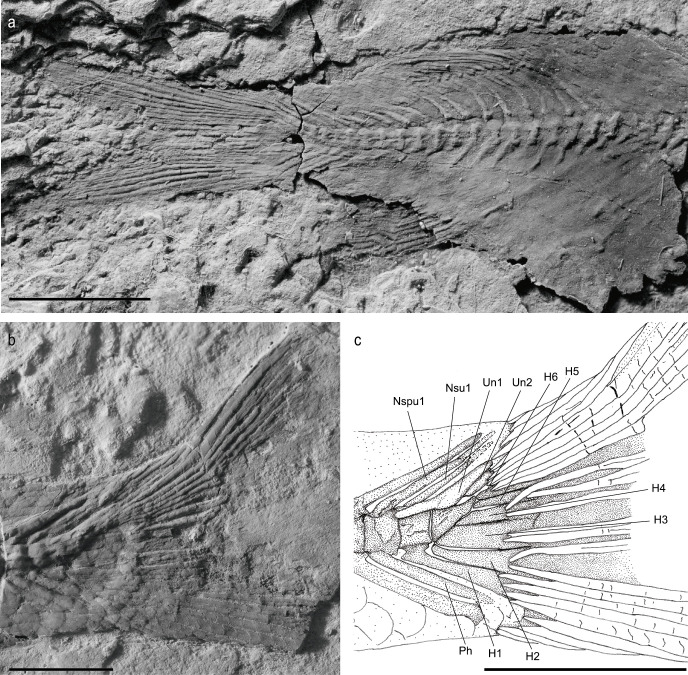
*Laeliichthys ancestralis* Santos, 1985 [1]. **A,** photograph of the specimen UERJ-DZ04. Scale bar equals 5 mm; **B,** photograph of the caudal region of UERJ-DZ03. Scale bar equals 5 mm; **C,** anatomical interpretations of the caudal endoskeleton. Abbreviations: H1–H6, first to sixth hypurals; Nspu1, neural spine on preural centrum 1; Nsu1, neural spine on Ural centrum 1; Ph, parhypural; Un1 and Un2, first and second uroneurals.

Both the dorsal and the anal fins are triangular and located posteriorly in the body (Figs 1A and 7A); the dorsal fin fits at the level of the 16th vertebrae and slightly anteriorly of the anal fin.

The dorsal fin has about four procurrent rays and twelve principal rays. The anal fin is slightly smaller than the dorsal fin. It consists of three procurrent rays and about ten principal rays. In both fins, the procurrent rays decrease in length posteriorly.

#### Vertebral column, caudal fin and skeleton

Thirty six or thirty seven well-ossified centra form the vertebral column. They are slightly deeper than long and have three to four longitudinal ridges on their lateral surface. The anterior three or four abdominal vertebrae do not bear ribs and are covered by the opercle and pectoral girdle. About 23 pairs of stout and long ribs, having similar proportions, extend to the ventral margin of the trunk and insert on the lower side of the abdominal vertebrae. There are 18 caudal centra, including the two ural centra. Some supraneural bones and thin longer epineurals are present posterior to the skull and associated with neural arches of all the abdominal vertebrae.

The caudal endoskeleton is partially preserved or is outlined below the scales. It comprises four distinct vertebral centra; two preural and two ural centra. The dorsal flexion of the rachis is clearly posterior to ural centrum 1. Preural centra 2 and 1 each carry a full length neural spine. The first ural centrum also has a complete neural spine dorsally.

Ventrally the parhypural presents the same dimensions as the previous haemal arches. Six hypurals can be observed; hypural 1 and hypural 2 each articulated seprately with ural centrum 1. Hypural 1 is larger and fan shaped while hypural 2 is more gently developed and its distal end is not fused to hypural 1. Hypural 3 is the largest one and shows a broad articulation with ural centrum 2. There are three upper hypurals that diminishes in length and thickness. Hypural 4 appears to articulate with the head of hypural 3, while hypurals 5 and 6 seem to lie free (Fig 7B and 7C).

There are two uroneurals. Uroneural 1 is the largest and reaches forward to cover the dorsolateral surfaces of Ural centrum 1 and preural 1. Uroneural 2 is simple and closely tied to the posterior margin of uroneural 1. It reaches as far as the posterior limit of ural centrum 2.

The caudal fin has a forked outline, with both lobes of equal size (Figs 2A and 7A). As usual in Osteoglossiformes, there are 18 principal caudal fin rays. There are 4 epaxial procurrent rays and 4 or 5 hypaxial procurrent rays.

#### Squamation

The body is entirely covered with large scales presenting fine circuli and radial furrows ([1]; Fig 2), a condition considered as plesiomorphic and also found in *Lycoptera*, hiodontids, notopterids, and *Joffrichthys* [38].

A continuous series of at least 37 lateral line scales extends from the head to the caudal peduncle. These scales decrease in height posteriorly. There are five lines of scales above and four or five under the lateral line. For a histological description of the scales see Meunier & Brito [26].

## Discussion

### Phylogenetic analysis

Since its original description, *Laeliichthys* has been recognized as an osteoglossomorph due to the presence of a well-developed basihyal tooth plate participating in the parasphenoid-tongue bite apparatus, among other characters. However, in the present study, the phylogenetic position of *Laeliichthys* is tested using a modified data matrix based on that of Wilson & Murray [37]. The first step of the analysis was a re-run of Wilson & Murray’s data matrix which confirm ed their results (six most parsimonious trees, with a length of 238, CI = 0.5084, HI = 0.4916, RI = 0.7388 and RC = 0.3475). The matrix was modified only by the inclusion of *Laeliichthys* (Table 1) and this inclusion did not change the interrelationships among the major clades of osteoglossomorphs obtained in that previous study.

**Table 1:** Character states for *Laeliichthys ancestralis* Santos 1985 [1].

?1100	232?1	100??	????1	11011	0?00?	?2002	10001	111??
?0???	????0	00101	10001	11200	102??	1?200	00???	?0

**Fig 8:**
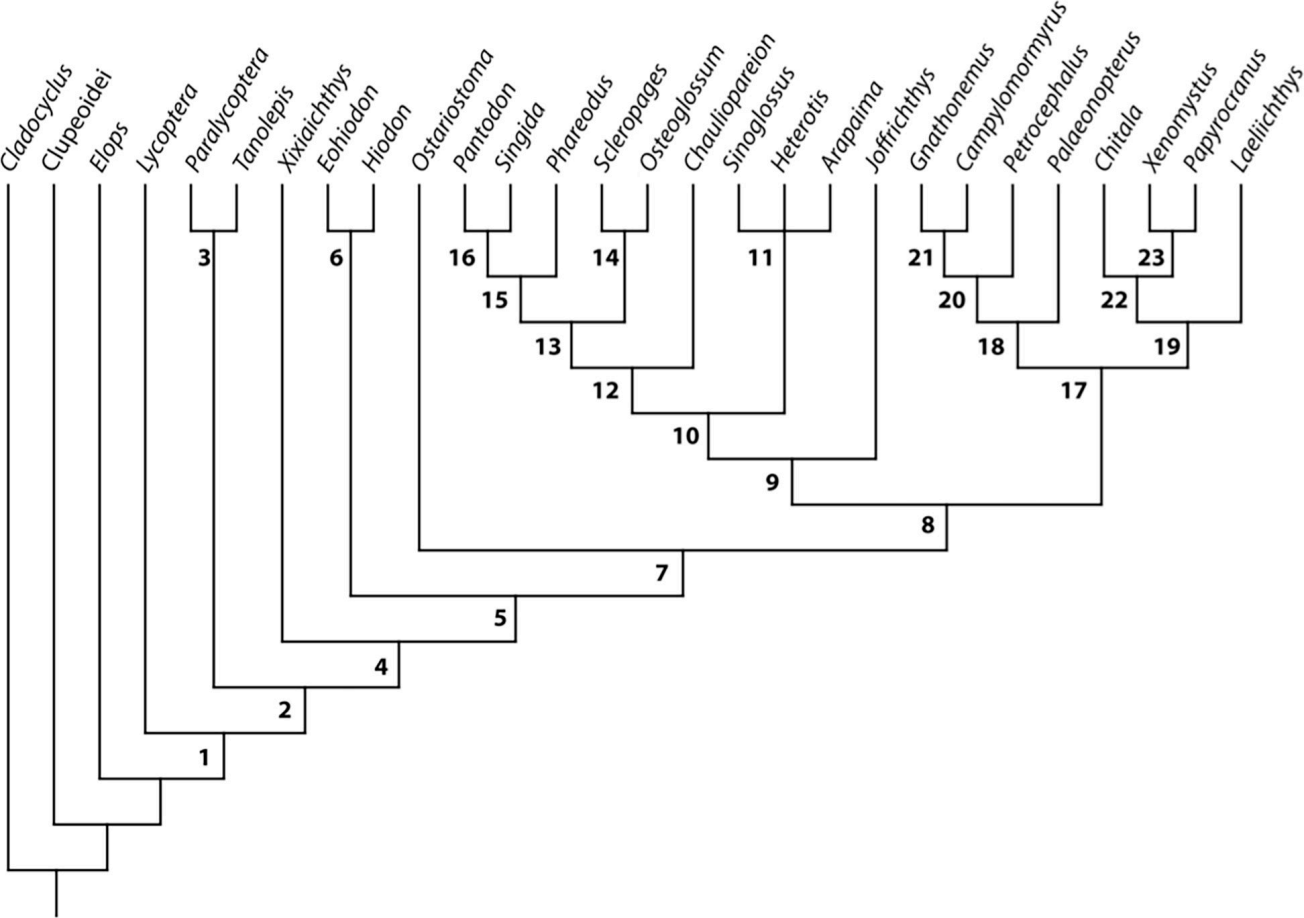
Strict consensus tree of the two equally most parsimonious trees (length 242, CI = 0. 5000, HI = 0.500, RI = 0.7381 and RC = 0.3690), analyzed with PAUP.

Two most parsimonious trees (MPTs) were obtained each with a tree length of 242 steps (CI = 0. 5000, HI = 0.500, RI = 0.7381 and RC = 0.3690). The resulting strict consensus tree displays a total of 23 components (Fig 8, S1 Fig). Bootstrap values were calculated in PAUP (1000 replicates of a heuristic search) (S2 Fig). The osteoglossomorphs (node 1) are confirmed as a monophyletic group.

Within this phylogenetic context, the Osteoglossiformes (Clade 8) contains two main nodes: Clade 9, formed by the Osteoglossoidei and clade 17, formed by the Notopteroidei. Clade 17 presents two main nodes: One, clade 18 formed by *Palaeonotopterus* (*Petrocephalus* (*Gnathonemus* + *Campylomormyrus*)), and the clade 19 (*Laeliichthys* (extant notopterids)) (Fig 8).

Most of the recovered relationships are similar to those recovered by Wilson & Murray [37]; the differences are mainly in the relationships between some of the osteoglossoids (Clade 9). In our hypothesis, the base of this clade differs significantly from that of [37] as *Joffrichthys* is here retrieved as the most basal taxon, followed by two clades: Clade 11 (*Sinoglossus*, *Heterotis*, *Arapaima*) and clade 12, with *Chauliopareion* as the sister taxon of clade 13. Our clade 13 (Fig 8) is formed by the modern osteoglossins and the node formed by *Phareodus (Pantodon + Singida)* [contra *Chauliopareion* (*Singida*, *Pantodon* (extant osteoglossins (*Phareodus* (*Joffrichthys* (*Heterotis*, *Arapaima*, *Sinoglossus*))))) of Wilson & Murray [37]]. As our main intention is to propose a hypothesis of phylogenetic position for *Laeliichthys*, here we only address the notopteroids of clade 17.

The Notopteroidei, resolved as Node 17 (Fig 8, S1 Fig), are well supported by two characters: mandibular canal open in a groove (cha. 41, ci = 1) and utriculus completely separated from sacculus and lagena (cha. 85, ci = 1). Seven other homoplastic synapomorphies support the monophyly of the notopteroids: temporal fossa present, with the exoccipital making a contribution to the border (cha. 1, ci = 0.600); nasal bones gutter-like (cha. 7, ci = 0.750); foramen for cranial nerve opens anterior to the prootic bridge (cha. 19, ci = 0.333); otic and supraorbital sensory canal partially or completely in grooves (cha. 21, ci = 0.500); opercle dorsal to facet for articulation with hyomandibula flattened or truncated (cha. 76, ci = 0.667); and second infraorbital expanded and equivalent in size to/or larger than third infraorbital(cha. 78, ci = 1).

*Laeliichthys* is unambiguously a member of the Notopteroidei because it presents: 1- the mandibular canal open in a groove; 2- the otic and supraorbital sensory canal partially or completely in grooves, homoplastic with *Pantodon*; 3- opercle shape, dorsal to facet for articulation with hyomandibula flattened or truncated, unknown in *Palaeonotopterus* and homoplastic with *Arapaima*; and 4- second infraorbital expanded and equivalent in size to or larger than third infraorbital, unknown in *Gnathonemus* and *Palaeonotopterus* among the notopteroids and homoplastic with *Tanolepis* and *Sinoglossus*.

A conspicuous synapomorphy of the notopteroids, also unknown in *Palaeonotopterus*, is that the nasal bones are gutter-like. Instead, in *Laeliichthys* the nasal bones are somewhat flat and broad, reminding those of *Chaulioparenion*, *Singida*, *Pantodon*, osteoglossins, *Phareodus*, *Joffrichthys*, and heterodontins (= arapaimins). However, contrary to all these taxa, in *Laeliichthys* the sensory canal, in the nasals, opens in a groove (Fig 6).

Within the notopteroids, *Laeliichthys* is rooted as the sister taxon of the monophyletic family Notopteridae (c.f. node 19, Fig 8). This pattern of relationship is supported by six shared derived characters: 1- extrascapular reduced and irregularly shaped (cha. 2, ci = 0.667); 2- nasal bones meeting each other in the midline (cha. 6, ci = 0.333), homoplastic with osteoglossins, *Joffrichthys*, *Sinoglossus*, and heterodontins (= arapaimins); 3- absence of subopercle (cha. 35, ci = 0.333), homoplastic with *Pantodon* and *Singida*; 4- a well-developed ascending process of the premaxilla (cha. 37, ci = 0.200), a state of character considered as plesiomorphic for osteoglossomorphs, reversed on notopterids + *Laeliichthys* and in *Pantodon*, *Phareodus*, *Joffrichthys*, *Signoglossus*, and *Arapaima*; 5- basihyal toothplate with ventrally directed processes (cha. 48, ci = 0.500), a derived character homoplastic with *Hiodon* and *Eohiodon*; and 6- Lateral line not piercing the supracleithrum (cha. 58, ci = 0.333), a state of character also found in *Ostariostoma*, osteoglossins and heterodontins.

#### Phylogenetic and paleobiogeographic implications

*Laeliichthys* has always been considered as an osteoglossiform, even before its formal description [17]. Santos [1] pointed numerous cranial and caudal similarities between *Laeliichthys* and *Heterotis*. However, he claimed that the ornamentation of the skull bones, the presence of a supramaxilla and the disposition of teeth in the vomer, parasphenoid, endopterigoid and basibranchial, as well as the difference in the number of vertebrae and the simplicity of the scales would be sufficient differences for the creation of a distinct subfamily, Laeliichthyinae, within the Arapaimidae (= Heterotidae).

Bonde [18] questioned the *Laeliichthys*—*Arapaima/Heterotis* synapomorphies, considering *Laeliichthys* to be a primitive osteoglossoid, plesiomorphic when compared to other osteoglossids (here including *Pantodon*). Li & Wilson [22], Li [23], and Li et al. [24] included *Laeliichthys* in phylogenetic analyzes, hypothesizing its position as a stem arapaimins. It is important to note that none of these authors had examined the specimens of *Laeliichthys* and that all the information used was based on Santos’ [1] original description.

Forey & Hilton [2] questioned the position of *Laeliichthys* as a stem arapaimine advocated previously as most of the evidences for this relationship was based on homoplasies. Nevertheless, these authors argued that an identification as an osteoglossid could be justified by the presence of two characters: a broad, flat nasal bone and the palatoquadrate area behind and below orbit completely covered by infraorbitals.

As discussed above, a broad and flat nasal bone is characteristic of crown osteoglossoids and *Xixiaichthys* [38]. *Laeliichthys* has flattened nasal bones that are slightly curved anteriorly. However, as in notopteroids, the sensory canal in these bones is partially open in a groove.

The palatoquadrate area behind and below orbit completely covered by infraorbitals was interpreted by Li et al. [24] and Hilton [38] as a synapomorphy of osteoglossoids, is not known in *Sinoglossus* and may be a possible reversion in *Chaulioparenion*. Due to the expansion of the second, third and fourth infraorbitals, *Laeliichthys* also presents this character state. The condition observed in *Laeliichthys* is, therefore, considered as homoplastic with that of the osteoglossoids.

Another character of importance relates to the scales. Hilton [38] recognised three states for osteoglossomorph’s scales: 1- with no reticulate furrows, 2- with both radial and reticulate furrows, and 3- with reticulate furrows present over the entire scale.

*Laeliichthys* shares with *Lycoptera*, hiodontids, notopterids, and *Joffrichthys* the plesiomorphic condition: scales with no reticulate furrows. Both the form and the histology of the *Laeliichthys´* scales were discussed by Meunier & Brito [26] who noted that this taxon, as well as notopterids, are devoid of *squamulae* and possess Mandl’s corpuscles within the scales. This condition had previously been recognized for *Hiodon* [47]. We did not examine any specimens of the genus *Joffrichthys*. However, the scales of this genus are cycloid [48,49] which, accepting its phylogenetic position, would represent the only osteoglossoid to possess this plesiomorphic condition. *Pantodon* and mormyrids possess both radial and reticulate furrows on their scales. However, in *Pantodon* the front of mineralization is relatively regular and structures very similar to the Mandl’s corpuscles can be seen [47]. Contrastingly, in mormyroids the scales lack Mandl’s corpuscles, a condition found only in taxa with reticulate furrows (e.g., osteoglossoids, excluding *Pantodon*).

The presence of cycloid scales is also strong evidence supporting the relationship between *Laeliichthys* and notopterids. In addition, the presence of squamules, similar to those of osteoglossoids and mormyrids, in the Anoual Formation, Bathonian (~166 Mya) of Morocco [9,50] push back to the Mid Jurassic the age of the osteoglossoids or the mormyrids (plus gymnarchids).

Our phylogenetic hypothesis in which *Laeliichthys* nests within Notopteroidei, is based on the synapomorphies cited above, mainly in the mandibular canal opening in a groove. Other homoplastic synapomorphies such as the otic and supraorbital sensory canal lying partially or completely in grooves, the shape of the opercle dorsal to the facet for articulation with hyomandibula, and the size of the second infraorbital are no less important. Simirarly, our results suggest a sister relationship between *Laeliichthys* and the Notopteridae (c.f., characters above).

The fossil record of notopterids is still very poor and restricted to otoliths from the Late Cretaceous Deccan Intertrappean Beds in India [51,52] and to a specimen from the Eocene of Sumatra [53] that appears to be closely related to the extant *Notopterus* [8,54]. The other clade within the freshwater Notopteroidei, the superfamily Mormyroidea has no reliable fossil record older than Miocene. It appears to be the sister group of the iconic taxon, *Palaeonotopterus greenwoodi* [37,45].

The notopteroids are represented today in Africa and Asia [54]. Previously, the most ancient record of Notopteroidei was *Palaeonotopterus* from the? Albian-Cenomanian Kem Kem Group of Morocco [45,55,56], providing a minimum age of ~94 Mya for the oldest presence of Notopteroidei in Africa. Using this fossil to calibrate the minimum age of Notopteroidei in a molecule-based study, Lavoué [57] estimated the mean age of the crown group Notopteroidei to 110 million years (95% credibility interval: 130–95 million years).

**Fig 9:**
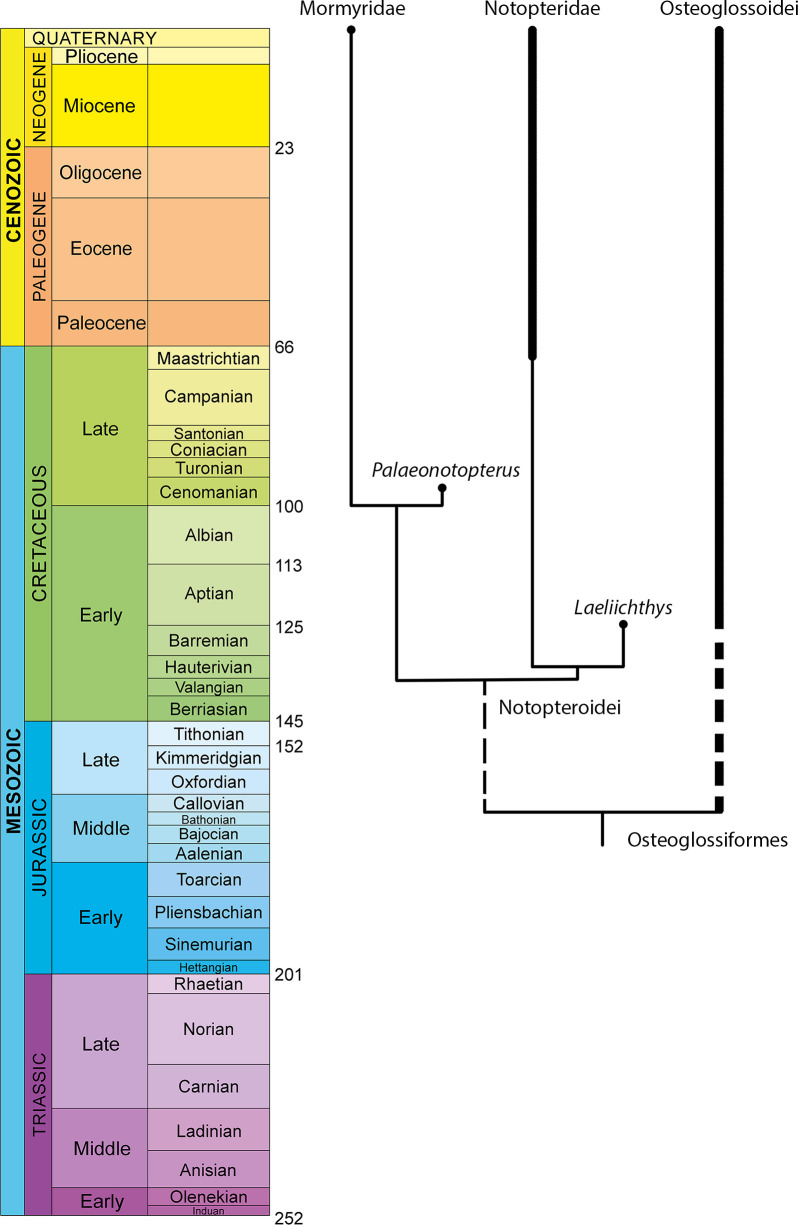
Simplified geological time range of notopteroids. Distribution through geological time modified from López-Arbarello (2012) [58] Thick lines represent sampled fossil record, while thin lines highlight the ghost lineages.

The phylogeny of Notopteroidei is still far from being completely resolved, especially regarding to the position of *Palaeonotopterus*. This is in partially due to the limited anatomical information available for this taxon which is known to date only by partial skulls and dental plates. Therefore, a better understanding of *Palaeonotopterus*, coupled with a revision of other African taxa, will be essential to determine the phylogenetic interrelationships within this clade. However, given that *Laeliichthys* is the sister taxon of notopterids, it can be assumed that this clade previously had a larger distribution, extending over western Gondwana (South America + Africa), before the final opening phase of the South Atlantic Ocean, giving a minimum Barremian age (121 Mya) for the group (Fig 9). Therefore, *Laeliichthys* extends the evolutionary origins of notopteroid lineages by at least ~27 Myr before their first appearance in the African fossil record (e.g. *Palaeonotopterus*).

## Conclusion

Based on new anatomical details, this study clearly demonstrates that *Laeliichthys ancestralis*, from the Quiricó Formation, in the Sanfranciscana Basin of Brazil, exhibits several characters that support its inclusion within the Notopteroidei. These include the mandibular canal open in a groove; the otic and supraorbital sensory canal partially or completely in grooves; the opercle shape; and the second infraorbital expanded and equivalent in size to or larger than third infraorbital.

Our results support the affinities between *Laeliichthys* and the Notopteridae, mainly based on the extrascapular reduced and irregularly shaped; nasal bones meeting each other in the midline; absence of subopercle; a well-developed ascending process of the premaxilla; basihyal toothplate with ventrally directed processes; and lateral line not piercing the supracleithrum.

*Laeliichthys* differs from other notopterids by presenting a combination of characters such as a nasal with semi-triangular outline, broad posteriorly with sensory canal opening in a groove; infraorbitals completely cover the palatoquadrate behind and below the orbit; and first pectoral fin ray enlarged and extremely long. The previous subfamily Laeliichthyinae is elevated to family rank.

Finally, *Laeliichthys* is the oldest known record of Notopteroidei, giving a minimum Barremian age (121 Mya) for the group. This data allows us to assume that this clade previously had a larger distribution, extending over western Gondwana, in what is now South America and Africa.

## Supporting information

S1 Fig Strict consensus tree of the two shortest trees for 28 taxa and 87 equally weighted characters showing the apomorphic list giving character changes for each nodes. Characters are based on Wilson and Murray (2008) [37].
(TIF)

S2 Fig Strict consensus tree of the two shortest trees (242 steps) for 28 taxa and 87 equally weighted characters showing the phylogenetic position of *Laeliichthys ancestralis* Santos, 1985, modified from Wilson and Murray (2008) [37]. Digits indicate percentage bootstrap support.
(TIF)
