# Supplementary figures and images for: A revision of Laeliichthys ancestralis Santos, 1985 (Teleostei: Osteoglossomorpha) from the Lower Cretaceous of Brazil: Phylogenetic relationships and biogeographical implications
Source: PLoS One. 2020 Oct 29;15(10):e0241009. doi: 10.1371/journal.pone.0241009 (PMC7595333; doi:10.1371/journal.pone.0241009)

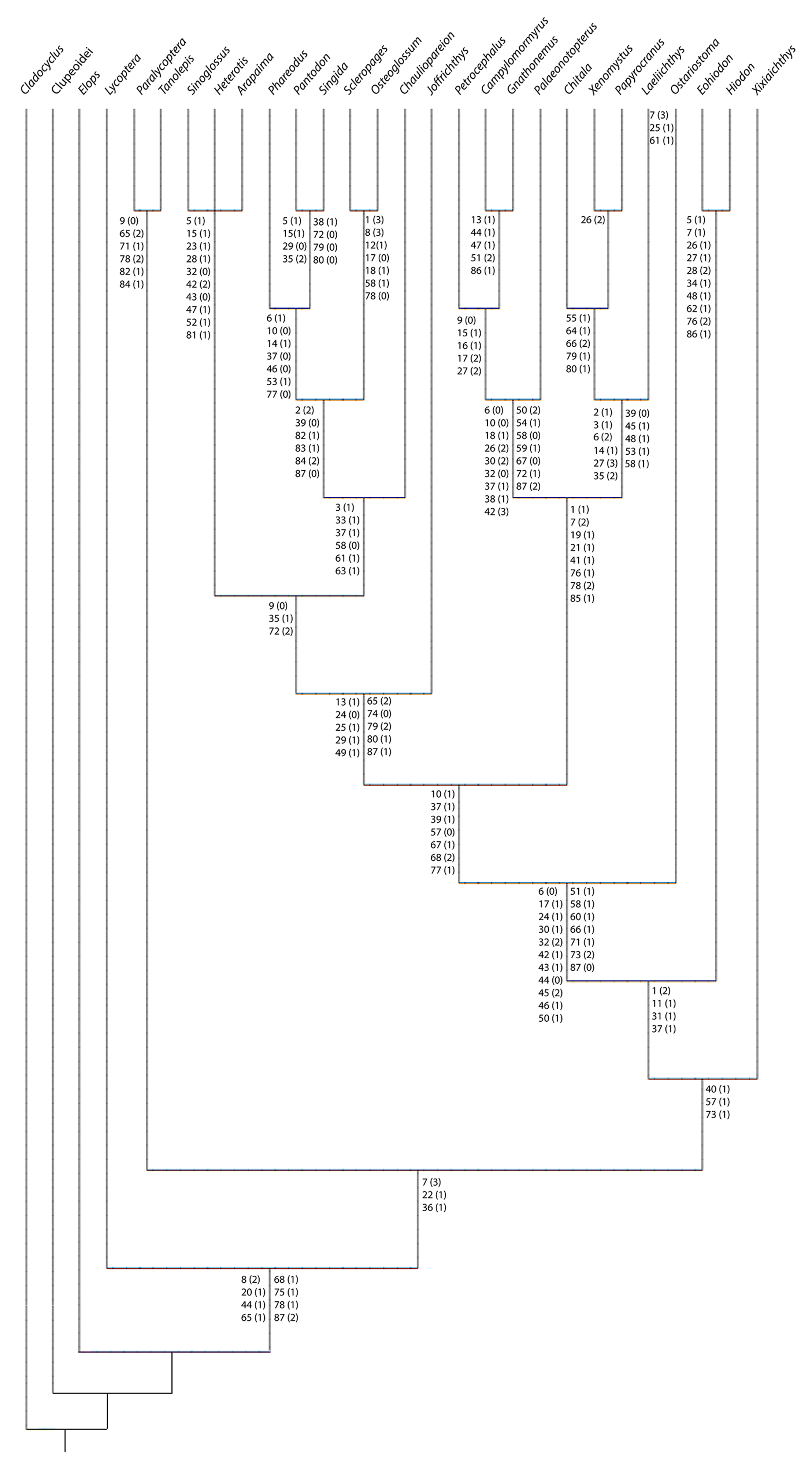

Supplement: S1 Fig — Characters are based on Wilson and Murray (2008) [37]. (TIF) [file pone.0241009.s001.tif]

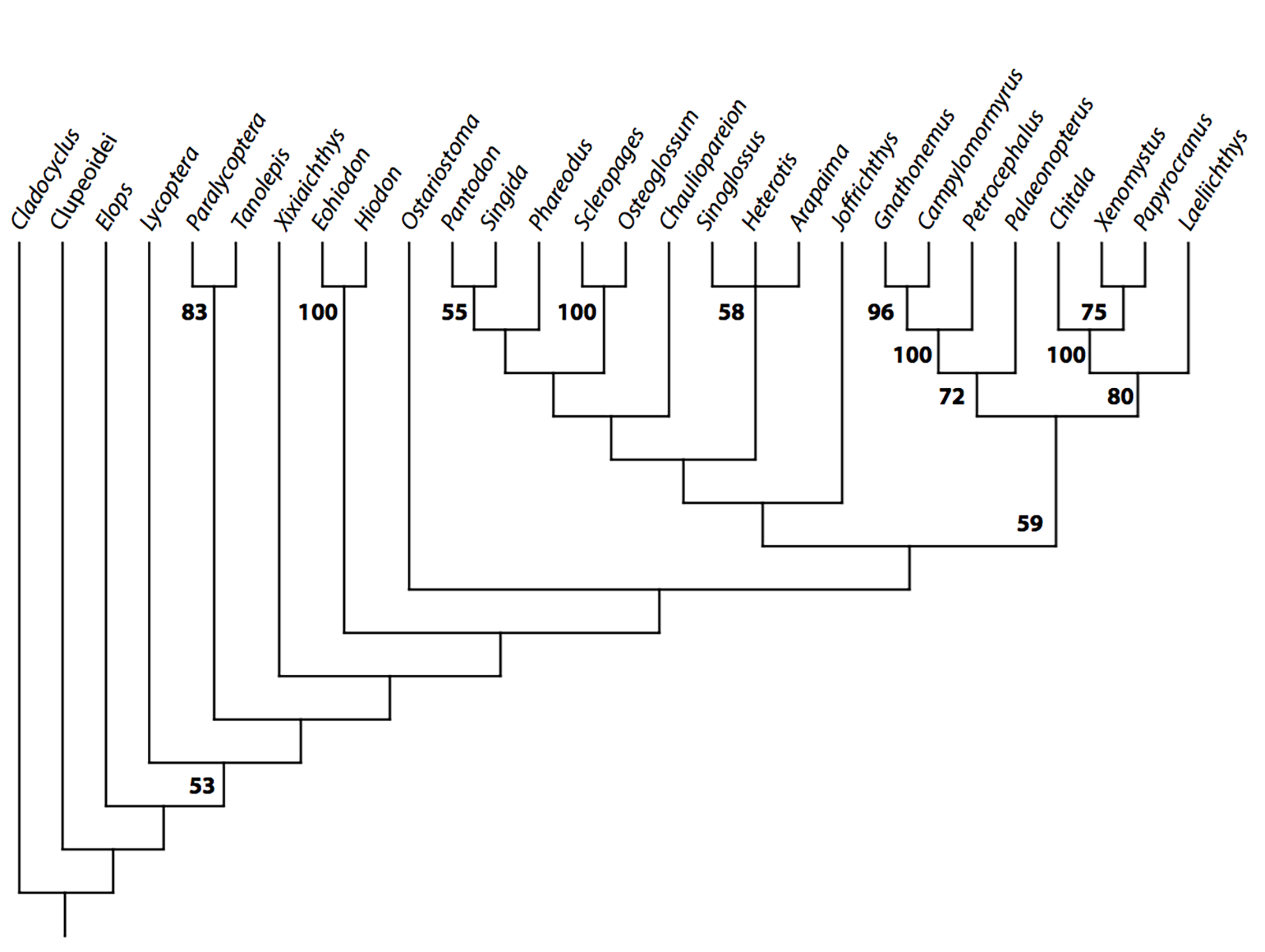

Supplement: S2 Fig — Digits indicate percentage bootstrap support. (TIF) [file pone.0241009.s002.tif]
